# On Public Influence on People’s Interactions with Ordinary Biodiversity

**DOI:** 10.1371/journal.pone.0130215

**Published:** 2015-07-08

**Authors:** Zina Skandrani, Lucie Daniel, Lauriane Jacquelin, Gérard Leboucher, Dalila Bovet, Anne-Caroline Prévot

**Affiliations:** 1 Centre d'Ecologie et des Sciences de la Conservation (CESCO UMR7204), Sorbonne Universités, MNHN, CNRS, UPMC, CP51, Paris, France; 2 Laboratoire d’Ethologie et Cognition Comparées (LECC), Université Paris-Ouest La Défense, Nanterre, Cedex, France; Landcare Research, NEW ZEALAND

## Abstract

Besides direct impacts of urban biodiversity on local ecosystem services, the contact of city dwellers with urban nature in their everyday life could increase their awareness on conservation issues. In this paper, we focused on a particularly common animal urban species, the feral pigeon *Columba livia*. Through an observational approach, we examined behavioral interactions between city dwellers and this species in the Paris metropolis, France. We found that most people (mean: 81%) do not interact with pigeons. Further, interactions (either positive or negative) are context and age-dependent: children interact more than adults and the elderly, while people in tourist spots interact more than people in urban parks or in railway stations, a result that suggests that people interacting with pigeons are mostly tourists. We discuss these results in terms of public normative pressures on city dwellers’ access to and reconnection with urban nature. We call for caution in how urban species are publically portrayed and managed, given the importance of interactions with ordinary biodiversity for the fate of nature conservation.

## Introduction

Mitigated success in preventing increasing biodiversity loss has been explained in recent years partly by growing individual disconnection from nature [1]. Pyle [2] explained this “extinction of experience” in urban areas as a cycle beginning with homogenization of biodiversity through habitat transformation, leading to impoverished human relations to nature, which in turn are further followed by even poorer environments and deeper isolation from nature. In this context of people’s estrangement from nature, raising broad-based public support for biodiversity conservation may become difficult [3]. This is particularly prominent in cities [4], where half of citizens worldwide are now living, a figure that is projected to reach 80% by 2050 [5]. Urbanites spend 90% of their time inside buildings [6] while nature-based recreation decreases [7].

Yet, besides formal environmental education, the success of conservation has been proposed to depend on people’s ability to experience biodiversity and maintain a direct connection with nature [8]. Given the high proportion of humans living in or near cities [9], restoring these essential human connections with natural elements depends massively on urban species and ecosystems, which are in closest proximity to where people live and work [4]. To improve environmental awareness among urban citizen, interaction with ordinary everyday nature should therefore be highly encouraged [10]. In most Western cities however, urban biodiversity is partly composed of species which have often negative cultural connotations, such as urban pigeons (*Columba livia*), House Sparrows (*Passer domesticus*), European Starlings (*Sturnus vulgaris*), cockroaches (*Blattaria*) and rats (*Rattus rattus*) [8]. These so-called “pest” species as well as habitats such as cracks between sidewalks and underpasses are commonly categorized as scary, unhealthy, and bad [11]. Interacting with these species as a way to open the door into a broader interest in wild nature appears therefore difficult and has therefore been termed the “pigeon paradox” [8]. Yet, these animal species are sometimes the easiest species to interact with, because of their abundance and/or familiarity with humans.

Given the increasing importance of city dweller’s interactions with ordinary biodiversity in cities [9], we were interested in the current paper in the factors that mediate these interactions in the urban landscape. Individual relations to nature are generally built in manifold interactions with several interrelated factors such as individual characteristics (age, personal history: [12]), the ecology of the species, the context of the interaction [13], but also public policies [14, 15]. In the current paper, we studied city dwellers’ interactions with urban pigeons, *Columba livia*, in Paris (France) and compared the relative effects of individual characteristics (i.e. age), location, and social context of interaction on the degree of these interactions.

Urban feral pigeons are one of the most common animal species in many western cities such as New York, London, Basle, Barcelona or Paris and are easy to interact with [16]. They are however often described as disease vectors even though few cases of disease transmission from pigeons to humans are reported in scientific studies [17], or as a source of nuisance for people ("flying rats", 11). In these large cities, pigeon populations are often managed [18] and a general feeding ban has been implemented to reduce the birds’ numbers [19].

We observed people-pigeons interactions in three different urban contexts in Paris (urban parks, local railway stations and touristic places), and asked the following questions:

First, do people interact more with urban biodiversity depending on the degree of nature in their environment? If yes, interactions with pigeons should be more important in places where nature is more present, i.e. urban parks.

Second, do people dedicate their interaction to biodiversity during more leisure oriented moments and activities? Then, interactions should be more important in urban parks and touristic places, where people can be expected to be more mindful than when going to work (suburban railway stations).

Third, are people’s interactions with biodiversity mediated by social considerations and public opinion? Then, Parisian citizen should be less prone to interact with pigeons, given the strong negative institutional campaign against them in Paris, than individuals that are away from their place of residence and own social context, i.e. tourists.

In addition, we compared interactions with pigeons of children, adults and elderly, in order to test for potential effects of age and gender.

## Materials and Methods

### 1. Data collection

We studied interactions between humans and pigeons in three spatially and socially different contexts in Paris (France), that are all highly frequented: two railway stations where city dwellers and suburb workers only pass through (Gare Montparnasse and Gare de Lyon), two urban parks that city dwellers generally use for recreation (Parc Montsouris and Jardin des Plantes) [20] and two tourist spots (Notre-Dame and Beaubourg). In each site, we focused our attention on a particular group of 5–20 randomly chosen pigeons and noted the behavior of people passing close to the flock. We defined each sampling site because of the regular presence of pigeons; in each sampling site, the group of pigeons we focused on remained present during the whole 2 hours duration of the sampling, even if pigeons individually moved and replaced each other.

Each sampling period lasted 2 hours, and was repeated 9 times in each site distributed in 3 week-days (consecutive or not depending on the site): in the morning (8:30–10:30), in the middle of the day (12:00–14:00) and in the afternoon (15:00–17:00). The data were collected from late April to late May 2010. This period was not a holiday period for Parisians; however it was the month with the second highest number of tourists that year with an average of over 3. 2 million [21]. Our touristic study sites are respectively the 1^st^ and 5^th^ most visited sites by tourists in Paris [21] and touristic predominance is easily observed by the abundance of tourist cars in these places. We thus assume that the people observed were mostly tourists.

Observations were conducted using the scan sampling method [22] of different human behaviors previously defined in a preliminary study (11 possible human behavior patterns towards pigeons, Table 1). Every 10 minutes during every 2-hour sampling period (i.e., 12 scan samplings per 2-hour sampling period), we scanned the behavior of the group of individuals present close to the pigeon flock and classified their behavior according to the 11 pre-defined behavioral categories. During the scan, people were also visually classified by gender and into three age groups: children (0–15 years old approximately), adults (16–60 years old approximately) and elderly people (more than 60 years old).

**Table 1:** Total numbers of observations (and proportions), according to the 11 pre-defined behaviors, two genders and three age-classes.

Behavior category	Behaviors	Women			Men		
		adults	children	Elderly	adults	children	elderly
Interactive	Walking quietly towards	5	16	2	9	30	1
	Observing	563	289	131	573	349	138
	Showing interest	75	15	6	71	13	10
	Feeding	57	60	18	57	84	37
	Being afraid	41	16	3	15	4	0
	Launching projectiles	0	0	0	2	5	0
	Making gesticulations	47	20	4	35	80	3
	Walking with dodging	11	10	3	16	2	4
	Run towards	13	92	2	17	209	0
	TOTAL INTERACTIVE	812 (17.1%)	518 (33.7%)	169 (14.1%)	795 (16.8%)	776 (43.1%)	193 (14.7%)
Neutral	Standing closeby without interest	1808	534	323	1665	459	368
	Walking without interest	2128	485	703	2284	567	751
	TOTAL NEUTRAL	3936 (82.9%)	1019 (66.3%)	1026 (85.9)	3949 (83.2%)	1026 (56.9%)	1119 (82.3%)

### 2. Definition of general behavior types

After collecting the data, we summarized the 11 pre-defined behavioral categories into 2 behavior types: neutral and interactive (see Table 1; S1 Dataset). Neutral behavior was defined as individual passing by or standing next to the group of pigeons without any interaction occurring. Interactive behaviors referred to individuals who looked at the pigeons, walked quietly towards them, showed an interest in them or fed them, individuals scared of the pigeons or trying to scare them, individuals walking around the group of pigeons and trying to dodge them, and individuals who ran towards pigeons with gesticulations.

In the subsequent analyses, we calculated the proportion of interactive behavior occurrences as equal to the number of interactive behavior occurrences divided by the total number of behavior occurrences (neutral and interactive).

### 3. Ethical notes

The study was based on anonymous observations only and excluded any kind of interactions with the study subjects. It was part of a multi-year research-action program on urban pigeons run by the French National Museum of Natural History (MNHN), for which permission to carry out studies in the public realm was granted by the city of Paris.

### 4. Statistical analyses

We first compared the proportions of interactive behavior occurrences among the three types of sites, the two genders and the three age categories, by using a generalized linear model with binomial error with the proportion of interactive behaviors as a dependent variable and the site types, age-classes and genders as fixed effects [23]. We controlled for the non-independence of data collected several times on the same site by including the site as a random factor in the model.

We compared the models by using Akaike Information Criterium (AIC), with two models being considered as significantly different whenever the difference in AIC values ΔAIC for the two models was higher than 2. We then tested the significance of each effect in the best model with a Student comparison with 0.

All the statistical analyses were done on R software [24], with the package lme4 [25].

## Results

We counted many less instances of interactive behavior than neutral behavior in all the situations: the percentage of neutral behavior averaged 81.6% (first quartile: 0.74 –last quartile: 0.95, Table 1). This indicates that the great majority of the people encountered did not interact with the pigeons at all.

**Fig 1:**
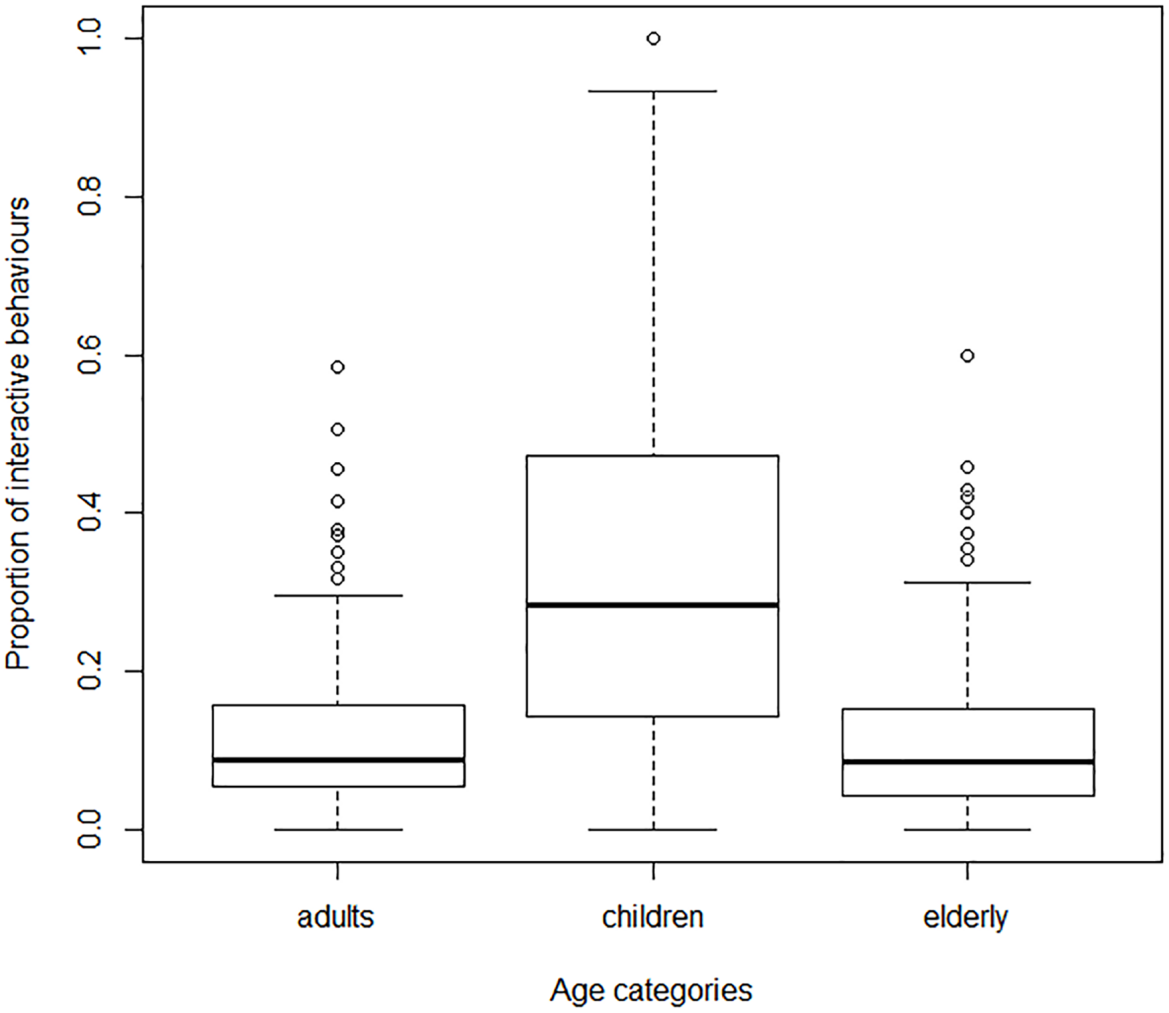
Proportions of interactive behaviours towards pigeons for three age categories.

**Fig 2:**
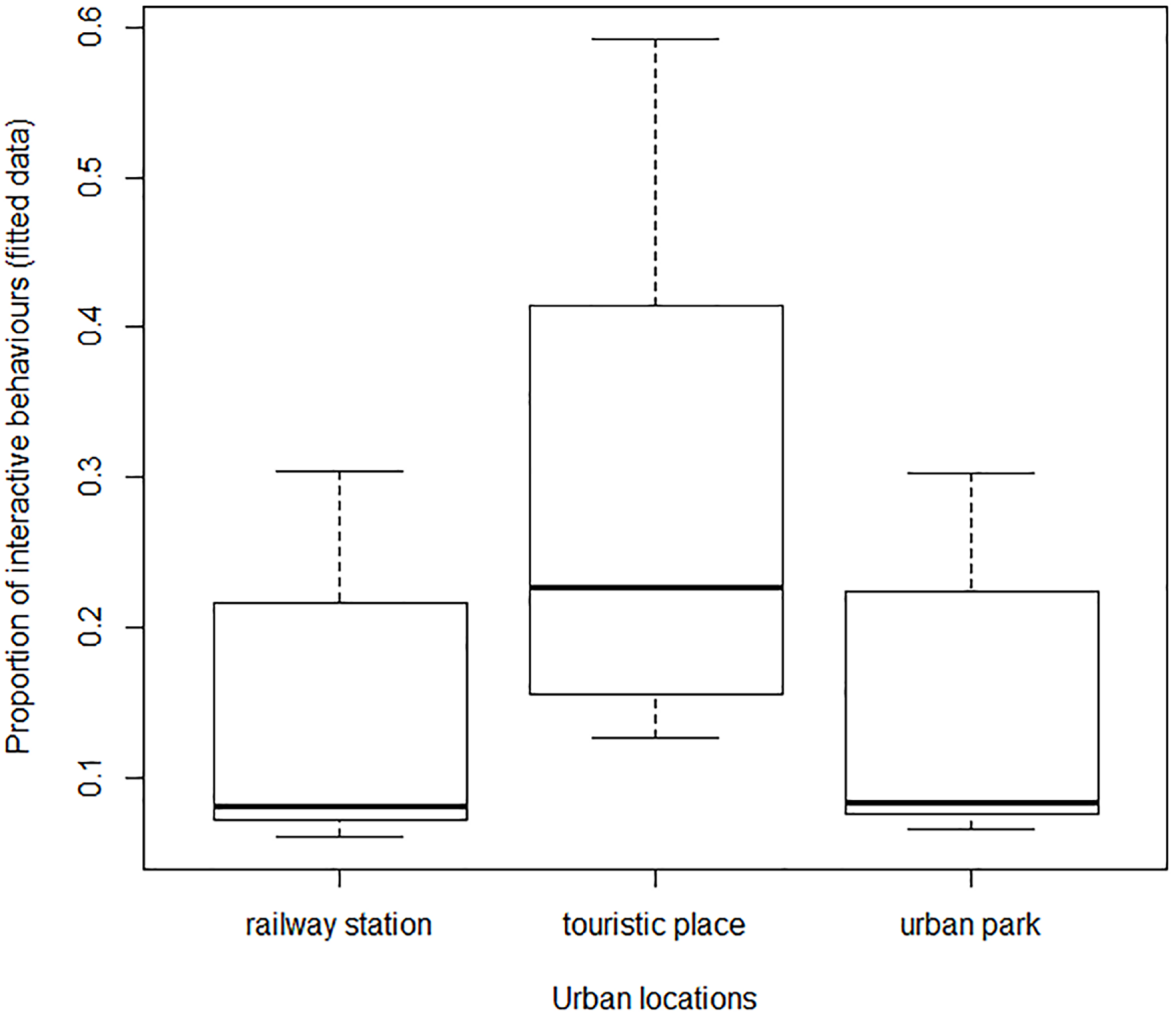
Proportions of interactive behaviours towards pigeons for three categories of urban places.

In more details, the proportion of interactive behaviors towards pigeons did vary between age categories, genders and locations (Table 2). If elderly people were significantly more neutral towards pigeons than adults (P = 0.001, Table 3), children were much more interactive towards pigeons than both adults (P<10^−10^, Table 3) and elderly people (Fig 1). According to gender, men and women did not differ in their neutrality towards pigeons except for children: boys were significantly less neutral than girls (P<10^−6^, Table 3). Finally, according to locations, the proportion of neutral behavior was significantly lower in the tourist spots than in either railway stations or urban parks (P<10^−8^, Table 3, Fig 2).

**Table 2:** Model selection based on AIC criteria.

Model	AIC
(1) Location + age + gender + age:gender + location:age + location:gender + location:age:gender	2529.28
(2) Location + age + gender + age:gender + location:age + location:gender	2526.74
(3) Location + age + gender + age:gender + location:gender	2527.83
**(4) Location + age + gender + age:gender + location:age**	**2523.02**
(5) Location + age + gender + location:age	2542.30
**(6) Location + age + gender + age:gender**	**2524.20**
(7) Location + age + gender	2543.36
(8) Age + gender + age:gender	2533.06

Age was modeled in three age categories (children, adults, elderly). Based on AIC, the two best models are presented in bold. For parsimony reasons, we selected the model (6) in our results.

**Table 3:** Estimates of each variable under selected model (model 6 in Table 2).

Variables	Estimate +- std error	Z-value	P-value
Location—touristic place	1.007 +- 0.173	5.837	5 e-09 ***
Location—urban park	0.038 +- 0.178	0.216	0.83 NS
Age—children	1.134 +- 0.065	17.353	< e-16 ***
Age—elderly	-0.255 +- 0.081	-3.157	0.00159 **
Gender—men	-0.029 +- 0.049	-0.596	0.55 NS
Children:men	0.416 +- 0.087	4.802	1. e-06 ***
Elderly:men	0.152 +- 0.111	1.372	0.17 NS

The control variables were respectively the railway stations for locations, adults for age and women for gender.

## Discussion and Perspectives

The overwhelming predominance of neutral behavior towards pigeons is the first main result of this study. Most children, as well as adults and elderly people, do not interact with this species and do not seem to notice their presence close by when they walk through the city. The result that city dwellers do not much interact with pigeons could be explained as the consequence of the cultural negative image of this particular species [11, 26]. However, it is also in line with other studies that have already indicated that city dwellers do not interact with urban nature in general [8; 1].

In more details, we found a diversity of these rare interactions, according to age and locations of encountering: children interact more with pigeons than older people, and more interactive behavior towards pigeons were observed in tourist spots than in either urban parks or railway stations. These results contradict our first hypothesis that people’s interactions with pigeons depend on the more or less natural character of their environment. They are neither consistent with our second hypothesis, that interactions with pigeons are more important in leisure times. However, the differences in interests towards pigeons, between children and adults or between touristic and non-touristic places, may be interpreted in terms of mindfulness, that is the receptive attention to and awareness of present experiences [27], characterized by a state of openness to novelty [28]. Though it has not been shown what encourages mindfulness in the touristic setting [29], we may hypothesize that in the touristic context, since it differs from everyday live environment, individuals have higher heightened attention towards their surrounding environment. In the same perspective, the higher interest of children towards pigeons may be explained by the higher curiosity of children towards nature [30], or a higher empathy of children towards animals compared to adults [31].

In addition to this individual-centered hypothesis, our results trigger further research questions about the contribution of social and institutional-level influence on the individual behaviors towards urban biodiversity. Consistent with other studies highlighting the impact of institutional communication and urban nature management on people’s perception of urban nature [32], it is interesting to consider our results under the perspective of normative pressures regarding pigeons.

Indeed, in order to mobilise action against a social problem, public service communicators often include normative information in their persuasive appeals [33]. In Paris, local authorities provided for several years normative messages on pigeons: first, pigeon (and other bird species) feeding is forbidden in public space from 1966 [26], for health reasons [19]; large communication campaigns used to be provided in urban parks, with both information flyers and fines. Second, pigeon populations are controlled since a long time by local authorities: historically by captures of adult pigeons, more recently by pigeon houses and control of pigeon reproduction [34]. Both feeding ban and so-called “contraceptive pigeon houses” provide normative messages to Parisian citizens, strongly suggesting than urban pigeons are too numerous, dangerous and maybe even “bad” species.

These public messages potentially act as injunctive norms, i.e. rules and standards of approved and disapproved conduct in the shared view of societal members, which have reported to have a substantial impact on individual actions, especially in environmental issues (littering, energy consumption, towel reuse in hotels) [35, 36, 37]. The extent to which a norm addresses a public behavior further increases the degree of conformity [38].

Under this perspective, our results of differential attitudes between local city-dwellers and tourists ask the question about the contribution of social validation and conformity pressure on people’s interaction with urban biodiversity. These normative considerations are indeed only influential in generating norm-consistent action within an in-group and among similar others [35, 39]. Yet individuals in the touristic setting are relieved of the primary costs of counter normative behavior e.g., social disapproval of their referent group or becoming social outliers [40].

Second, the potential role of norms as behavioral guides is moderated by situational factors. For instance, norms influence behavior only when they are activated, i.e. when the norm is made focal in consciousness at the time of the behavioral act [37]. If there is no salience, behavior is largely unguided by normative considerations [35, 33]. Here, the pigeon avoidance norm invoked for local Parisians through printed messages spread over the city, could be not primed for tourists who do not read French. Hence, even if familiar with similar social norms against pigeons in their home countries, tourists may be not focused on this norm in the touristic context.

In the light of our results and the importance of interaction with ordinary biodiversity for the fate of nature conservation [4, 10], we call for further research investigating more precisely the costs and benefits linked to how public policies portray and manage non-native and so-called pestiferous urban species.

## Supporting Information

S1 Dataset People’s neutral and interactive behaviours towards pigeons. (XLSX)
